# The risk factors for postoperative cerebral complications in patients with Stanford type a aortic dissection

**DOI:** 10.1186/s13019-019-1009-5

**Published:** 2019-10-22

**Authors:** Yong Lin, Mei-Fang Chen, Hui Zhang, Ruo-Meng Li, Liang-Wan Chen

**Affiliations:** Department of Cardiovascular Surgery, Fujian Medical University Union Hospital, No. 29 Xinquan Road, Fuzhou City, 350001 Fujian Province People’s Republic of China

**Keywords:** Postoperative complications, Circulatory arrest, deep hypothermia induced, Oximetry, Aneurysm, dissecting

## Abstract

**Background:**

Postoperative cerebral complications (PCC) are common and serious postoperative complications for patients with Stanford type A aortic dissection (AAD). The aim of this study was to evaluate the risk factors for PCC in these patients and to provide a scientific basis for effective prevention of PCC.

**Methods:**

In this retrospective case-control study, 125 patients with AAD who underwent thoracotomy in our department from October 2017 to October 2018 in the department of cardiovascular surgery, Fujian Medical University Union Hospital were divided into two groups: patients with PCC (*n* = 12), and patients without PCC (*n* = 113). The general clinical data, the types of corrective surgeries, the intraoperative situations, the postoperative complications, and the midterm outcomes of the patients were analyzed.

**Results:**

The patients with PCC were significantly older than the patients without PCC (*P* = 0.016), and the incidence of the preoperative cerebral disease history in the patients with PCC was significantly higher than those of the PCC (−) group (*P* = 0.024). The Euro SCORE II of patients with PCC was dramatically higher than the patients without PCC (*P* = 0.005). There were significant differences between the two groups in terms of the duration of cardiopulmonary bypass (CPB) (*P* = 0.010) and the length of moderate hypothermic circulatory arrest (MHCA) combined with selective cerebral perfusion (SCP) (*P* = 0.000). The monitoring of rcSO_2_ indicated that there was significant difference between the two groups in terms of the bilateral baseline (*P* = 0.000). Patients with PCC were observed to have experienced significantly longer intubation times (*P* = 0.000), ICU stays (*P* = 0.001), and postoperative hospital stays (*P* = 0.009), and they also had dramatically higher rates of pulmonary infection (*P* = 0.000), multiple organ dysfunction syndrome (*P* = 0.041) and tracheotomy (*P* = 0.022) after surgeries. The duration of MHCA+SCP (OR:9.009, *P* = 0.034) and the average baseline value of rcSO_2_ (OR:0.080, *P* = 0.009) were ultimately identified as significant risk factors.

**Conclusions:**

PCC has a serious influence on the prognoses of patients following surgical treatment with AAD. The duration of MHCA+SCP and the average baseline value of rcSO_2_ were the independent risk factors for PCC.

## Background

Postoperative cerebral dysfunction is the most common complication after the surgery for Stanford type A aortic dissection (AAD), and it has a negative effect that cannot be ignored in the postoperative rehabilitation of patients with AAD. Although the intraoperative cerebral protection strategy has been modified in recent decades, the morbidity due to postoperative cerebral complications (PCC) have been reported to be 6.4%~ 16.9% in cardiovascular surgical centers around the world [1–3]. Therefore, it is extremely important for cardiovascular surgeons and ICU physicians to explore the pathogenesis of PCC. The aim of this study was to evaluate the risk factors of postoperative cerebral dysfunction in these patients and to provide a scientific basis for the effective prevention of PCC.

## Methods

**Fig. 1:**
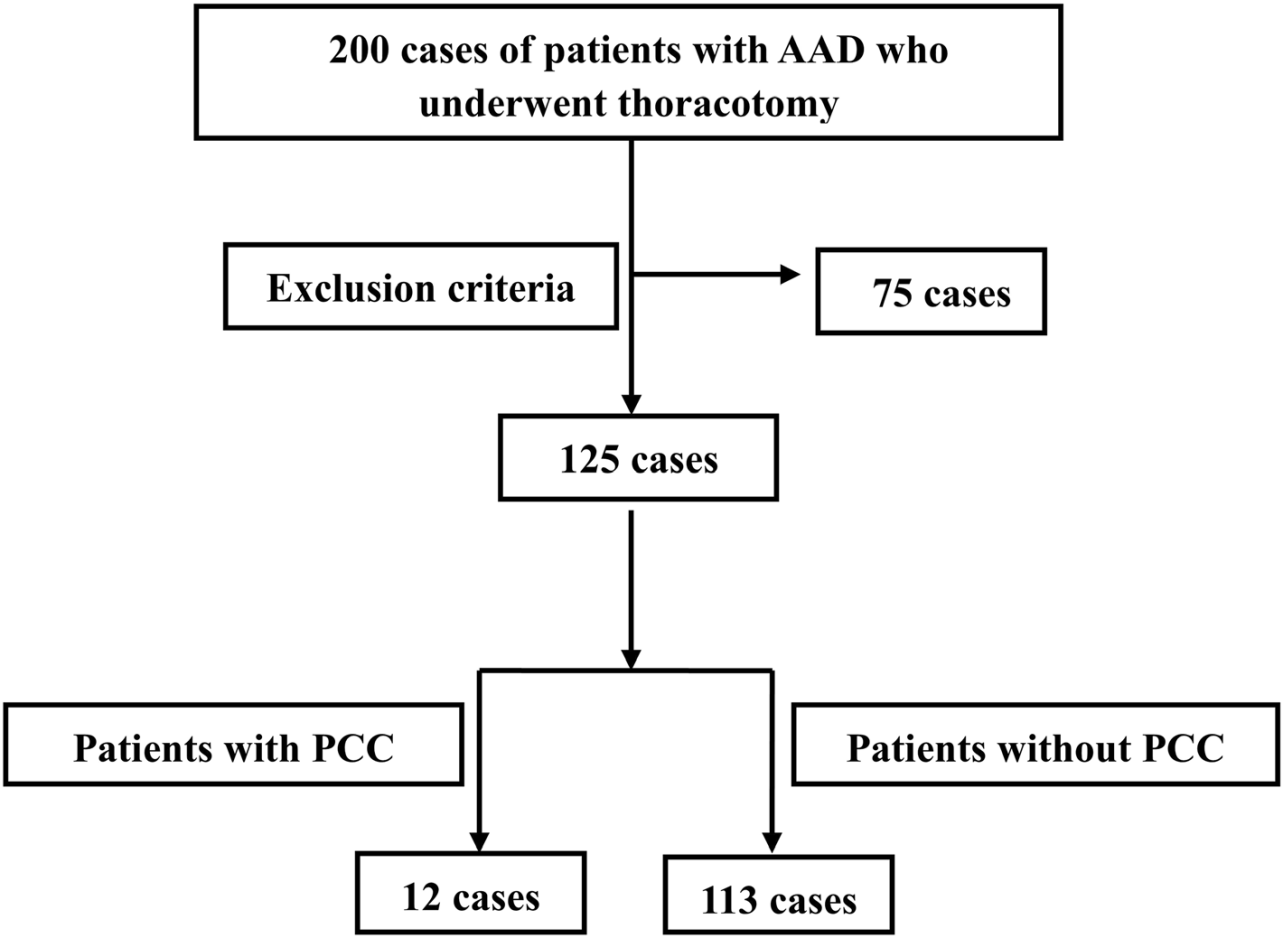
Flow Chart. One hundred and twenty-five patients with Stanford type A aortic dissection who underwent thoracotomy were selected from 200 patients based on the exclusion criteria, and they were divided into two groups: patients with PCC (*n* = 12), and patients without PCC (*n* = 113). Seventy-five patients were excluded from this study according to the exclusion criteria. AAD: Stanford type A aortic dissection; PCC: postoperative cerebral complications

Two hundred consecutive patients with AAD who underwent thoracotomy in our department from October 2017 to October 2018 were enrolled in this retrospective case-control study. Then, the patients were divided into two groups: patients with PCC and patients without (Fig. 1). The general clinical data, types of corrective surgeries, intraoperative situations, postoperative complications, and midterm outcomes (12 months) of the patients were analyzed by telephone follow-up or out-patient review.

### Exclusion criteria

1. Patients who were younger than 18 years old.
2. Patients who were died before surgeries.
3. Patients with an abnormal preoperative consciousness.
4. Patients and/or their relatives who did not agree to participate in this clinical study.
5. Patients who were lost within the 12-month follow-up period.

### Diagnostic criteria of the postoperative cerebral complications

The PCC included new-onset stroke, syncope, delirium, postoperative cognitive dysfunction (POCD), delayed emergence from anesthesia (DEA) and coma. The diagnosis of a stroke was based on the National Institutes of Health Stroke Scale (NIHSS) [4]. Syncope was defined as a transient loss of consciousness and was characterized by a rapid onset, short duration, and spontaneous complete recovery [5]. The confusion assessment method for the intensive care unit (CAM-ICU) [6] was applied for the evaluation of postoperative delirium (POD) and POCD. The Glasgow Coma Scale (GCS) [7] was used to objectively define coma in the two groups of patients after their surgeries. The patients whose response to stimulation occurred more than 60~90 min after the surgeries were recognized as having a delayed emergence from anesthesia [8], and this diagnosis should exclude the possibility of the other types of cerebral dysfunction mentioned above.

All of the diagnoses of PCC were confirmed by two experienced neurologists. Deeply sedated patients, confirmed by the monitoring sedation status system on the Richmond Agitation Sedation Scale (RASS) [9], did not receive an evaluation of cerebral function until they recovered from anesthesia.

### Protocols of anesthesia

Combined intravenous-inhalation anesthesia was applied in the patients. The nasopharyngeal temperature and the rectal temperature were also monitored. Transesophageal echocardiography (TEE) was applied to monitor the intraoperative hemodynamics. Autologous blood transfusion was used to reduce the need for allogeneic blood transfusion. The balances of cerebral oxygen metabolism of the patients were measured by regional cerebral oxygen saturation (rcSO_2_) with the Regional Oximetry System (INVOS™ 5100C, Medtronic, USA). The bispectral index (BIS), which was measured by the BIS Monitoring System (VISTA, USA), was used to measure the depth of anesthesia.

Mechanical ventilation was performed after endotracheal intubation and was applied to provide end tidal CO_2_ volumes (PetCO_2_) of 35~40 mmHg, a FiO_2_ of 50~100%, a tidal volume of 6~8 ml/kg, a respiratory rate of 10~12 breaths/min and an adequate positive end-expiratory pressure (PEEP). The maintenance of anesthesia was implemented by an intravenous injection of 4~8 mg/kg/h propofol and 1~2 μg/kg/h sufentanil along with intermittent intravenous injections of cisatracurium.

During the surgery, the values of scSO_2_ were maintained at or above 70% of the baseline threshold. Cerebral desaturation was defined as a decrease in the saturation value below the absolute value of 50% or 70% of the baseline for 15 s. The mean and minimum values of the rcSO_2_, as well as the area under the curve (AUC) of the rcSO_2_ values below the line of the previously mentioned cerebral desaturation values, were collected for further analysis.

### Surgical procedures

During surgery, A sternal incision was performed. To establish cardiopulmonary bypass (CPB), the arterial cannula was placed in the right femoral artery and/or the right axillary artery, and the drainage tube was placed in the right atrium. The CPB flow rate was 2.4~2.6 L/kg/min. The intermittent cold blood cardioplegia was perfused through the left and right coronary arteries for myocardial protection. The following procedures, including the triple-branched stent graft implantation [10] and the Sun’s procedures were described in the previous literature [11].

### Protocol to address intraoperative cerebral desaturation

The positions of the patients were checked first to exclude the compression of the cervical great vessels when the intraoperative cerebral desaturation occurred. Then, the parameters of the mechanical ventilator (before or after CPB) or the oxygenator (during CPB) were adjusted to maintain the arterial pressure of CO_2_ (PaCO_2_) above 40 mmHg. Metaraminol or noradrenaline was used to elevate the mean arterial pressure (MAP) to above 60 mmHg. A cardiotonic was administered if there was a poor cardiac index (below 2.0 L/m^2^/min), and adequate blood volume was confirmed by TEE. The BIS value was maintained at or below 50 during the CPB to ensure that patients were in a state of deep anesthesia. Other methods to prevent cerebral hypoxia included increasing the pump flow, increasing the FiO_2_ and performing allogeneic blood transfusion.

### Follow-up

Telephone contact or out-patient review with the patients was maintained after discharge. Every month in the first year, the patients received echocardiographies, chest radiographies, and bilateral carotid artery Doppler examinations. At 1 month and 3 months after surgery, the patients received aortic computed tomography angiography (CTA) examinations, which were then performed annually.

### Statistical analysis

SPSS software (version 19.0, IBM, USA) was used for the statistical analyses. Descriptive statistical analyses, as well as Wilcoxon rank sum tests, were used to analyze the measurement data. The chi-square test or Fisher’s exact test were used to analyze the numerical data. Multivariate logistic regression analysis was used to analyze the count data. Statistical significance was defined as *P* < 0.05.

## Results

### General clinical data

Seventy-five patients were excluded from this study after meeting the exclusion criteria described above, including 9 cases who were younger than 18 years old, 42 cases with an abnormal preoperative consciousness, 8 cases died before surgeries, 13 cases who did not agree to participate in this clinical study, 3 cases who were lost within the 12-month follow-up period. One hundred twenty-five cases were included after applying the exclusion criteria. Then, the patients were divided into two groups: patients with PCC (*n* = 12) and patients without (*n* = 113).

The primary analyses revealed that the patients in the PCC (+) group were significantly older than the patients in the PCC (−) group (58.4 ± 8.4 years vs 49.4 ± 13.4 years, *P* = 0.016), and the incidence of preoperative cerebral disease history in the patients of the PCC (+) group was significantly higher than that of the PCC (−) group (16.7% vs 0.9%, *P* = 0.024). The Euro SCORE II of patients in the PCC (+) was dramatically higher than the patients of the PCC (−) group (11.9 ± 2.7 vs 9.0 ± 3.4, *P* = 0.005). There were no significant differences between the patients in the two groups in terms of gender, body mass index (BMI), personal history, underlying diseases (except for preoperative cerebral disease history), New York Heart Association (NYHA) class, etiologies, ultrasound cardiogram (UCG) results, preoperative complications due to aortic dissection, scale of aortic dissection and American Society of Anesthesiologists (ASA) class (Table 1).

**Table 1:** Clinical data

Category of clinical data	PCC (+) *n* = 12	PCC (−) *n* = 113	*P*
Age	58.4 ± 8.4	49.4 ± 13.4	0.016
Gender			1.000
Male	11 (91.7%)	96(85.0%)	
Female	1(8.3%)	17(15.0%)	
BMI	24.1 ± 2.8	24.4 ± 3.3	0.987
Active smoking	6(50.0%)	48(42.5%)	0.761
Alcoholism	1(8.3%)	11(9.7%)	1.000
Underlying diseases			
Diabetes	0(0.0%)	2(1.8%)	1.000
CAD	0(0.0%)	1(0.9%)	1.000
Cardiac reoperation	1(8.3%)	2(1.8%)	0.263
Renal dysfunction	1(8.3%)	3(2.7%)	0.336
History of cerebral diseases	2(16.7%)	1(0.9%)	0.024
History of anemia	1(8.3%)	7(6.2%)	0.565
NYHA class			
I	0(0.0%)	29(25.7%)	0.078
II	10(83.3%)	65(57.5%)	
III	1(8.3%)	16(14.2%)	
IV	1(8.3%)	3(2.7%)	
Etiologies			
Hypertension	7(58.3%)	87(77.0%)	0.291
Others	5(41.7%)	26(23.0%)	
UCG			
EF (%)	61.6 ± 8.3	60.8 ± 7.9	0.725
Pericardial effusion^a^	0(0.0%)	5(4.4%)	1.000
Aortic regurgitation^b^	1(8.3%)	17(15.0%)	1.000
Preoperative complications			
AMI^c^	1(8.3%)	4(3.5%)	0.402
Lower limb ischemia	0(0.0%)	16(14.2%)	0.361
Mesenteric artery infarction^d^	0(0.0%)	7(6.2%)	1.000
The scale of aortic dissection			
Ascending aorta	1(8.3%)	4(3.5%)	0.402
Aortic arch	5(41.7%)	48(42.5%)	1.000
Innominate artery	5(41.7%)	23(20.4%)	0.138
Right common carotid artery	4(33.3%)	14(12.4%)	0.071
Left subclavian artery	2(16.7%)	10(8.8%)	0.323
ASA status			
I	0(0.0%)	0(0.0%)	1.000
II	0(0.0%)	0(0.0%)	
III	0(0.0%)	0(0.0%)	
IV	11(91.7%)	10.5(92.9%)	
V	1(8.3%)	8(7.1%)	
VI	0(0.0%)	0(0.0%)	
Euro SCORE II	11.9 ± 2.7	9.0 ± 3.4	0.005

The results demonstrated that the patients in the PCC(+) group were significantly older than the patients in the PCC(−) group, and the incidence of the preoperative cerebral disease history in the patients of the PCC(+) group was significantly higher than those of the PCC(−) group. The Euro SCORE II of patients in the PCC(+) group was dramatically higher than the patients of the PCC(−) group

*PCC* Postoperative cerebral complications, *BMI* Body mass index, *CAD* Coronary artery disease, *NYHA* New York Heart Association, *UCG* Ultracardiography, *EF* Ejection fraction, *AMI* Acute myocardial infarction, *ASA* American Society of Anesthesiologists

^a^serious pericardial effusion; ^b^ serious aortic regurgitation; ^c^ clinical manifestations, ECG, and contents of creatine kinase and troponin in the serum that were consistent with the diagnostic criteria for acute myocardial infarction; ^d^ confirmed by superior mesenteric artery angiography

### Surgical and perioperative treatments

The chi-square test revealed that there were no significant differences in the types of surgical procedures between the patients in the two groups. The Wilcoxon rank sum tests also indicated that there were significant differences between the two groups in terms of the duration of CPB (165.4 ± 42.5 min vs 134.8 ± 21.5 min, *P* = 0.010) and the length of moderate hypothermic circulatory arrest combined with selective cerebral perfusion (MHCA + SCP) (20.3 ± 2.1 min vs 15.5 ± 2.7 min, *P* = 0.000). The monitoring of rcSO_2_ indicated that there were no significant differences between the two groups in terms of intraoperative rcSO_2_ except for the average value of bilateral rcSO_2_ at baseline (48.2 ± 3.3% vs 66.7 ± 11.7%, *P* = 0.000). However, the duration of surgery, aortic cross-clamping, the mean value of the intraoperative BIS index, the volumes of blood loss and perioperative allogeneic transfusion were not significantly different in the patients in these two groups (Table 2).

**Table 2:** Surgical and perioperative treatments

Category of perioperative treatments	PCC (+) *n* = 12	PCC (−) *n* = 113	*P*
Types of surgical correction			
Aortic sinus reconstruction	3(25.0%)	17(15.0%)	0.406
Bentall	1(8.3%)	11(9.7%)	1.000
Wheat	0 (0.0%)	2(1.8%)	1.000
Hemiarch replacement	12(100%)	106(93.8%)	1.000
Ascending aorta replacement	12(100%)	105(92.9%)	1.000
CABG	0(0.0%)	1(0.9%)	1.000
Intraoperative conditions			
Surgery (min)	303.6 ± 43.0	282.4 ± 55.9	0.113
CPB (min)	165.4 ± 42.5	134.8 ± 21.5	0.010
Aortic cross-clamping (min)	50.8 ± 7.6	46.8 ± 7.9	0.064
MHCA+ SCP (min)	20.3 ± 2.1	15.5 ± 2.7	0.000
Blood loss (ml)	475.0 ± 256.3	374.8 ± 166.5	0.059
Avg rcSO_2_ baseline (%)^a^	48.2 ± 3.3	66.7 ± 11.7	0.000
Avg rcSO_2_ (%)(L)	56.5 ± 8.2	58.7 ± 9.9	0.505
Avg rcSO_2_ (%)(R)	57.3 ± 7.1	59.6 ± 11.7	0.560
rcSO_2_ minimum (%)	50.6 ± 7.8	54.7 ± 7.3	0.077
Total time of rcSO_2_ < 70% baseline and > 15 s (n)	4.6 ± 1.8	3.6 ± 2.4	0.174
Total time of rcSO_2_ < 50% and > 15 s (n)	1.7 ± 1.0	1.3 ± 1.6	0.099
AUC of rcSO_2_ < 70% (%min)(L)	150.3 ± 63.0	121.2 ± 75.5	0.227
AUC of rcSO_2_ < 70% (%min)(R)	120.3 ± 117.0	98.5 ± 63.5	0.917
AUC of rcSO_2_ < 50% (%min)(L)	86.6 ± 62.7	61.4 ± 64.1	0.145
AUC of rcSO_2_ < 50% (%min)(R)	52.7 ± 32.3	45.9 ± 49.7	0.303
Avg BIS index	36.8 ± 10.3	39.8 ± 9.3	0.320
Perioperative allogeneic transfusion			
RBC (u)	2.7 ± 1.8	3.1 ± 2.8	0.727
PLT (u)	0.8 ± 1.2	1.2 ± 1.9	0.201
FFP (ml)	366.7 ± 602.0	313.7 ± 345.8	0.666
CP (U)	1.0 ± 2.3	1.5 ± 2.8	0.528

The results demonstrated that there were significant differences between the two groups in terms of the duration of CPB and MHCA+SCP. The monitoring of rcSO_2_ indicated that there were no significant differences between the two groups in terms of intraoperative rcSO_2_ except for the average value of bilateral rcSO_2_ at baseline

*PCC* Postoperative cerebral complications, *CABG* Coronary artery bypass grafting, *CPB* Cardiopulmonary bypass, *MHCA* Moderate hypothermic circulatory arrest, *SCP* Selective cerebral perfusion, *rcSO*_*2*_ regional cerebral oxygen saturation, *AUC* Area under the curve, *RBC* Red blood cell, *PLT* Platelet, *FFP* Fresh frozen plasma, *CP* Cryoprecipitation

^a^the baseline value of rcSO_2_ from the bilateral brain before anesthesia induction

### Postoperational situation

Twelve (12/125, 9.6%) patients with postoperative cerebral complications were observed. The Wilcoxon rank sum tests showed that the anesthesia recovery periods of the patients in the PCC (+) group were shorter than those of the patients in the PCC (−) group, but there was no significant difference. Two cases of newly onset stroke confirmed by computed tomography (CT) or magnetic resonance imaging (MRI) (2/125, 1.6%), 1 case of syncope (1/125, 0.8%), 2 cases of POD (2/125,1.6%), 3 cases of POCD (3/125, 2.4%), 2 cases of DEA (2/125, 1.6%), 2 cases of coma (2/125, 1.6%) and 2 cases of postoperative paraplegia were observed in this study. Patients from the PCC (+) group were observed to have experienced significantly longer intubation times (69.3 ± 28.8 h vs 33.3 ± 24.2 h, *P* = 0.000), ICU stays (127.3 ± 72.0 h vs 63.5 ± 51.3 h, *P* = 0.001), and postoperative hospital stays (32.0 ± 16.7 d vs 21.2 ± 13.0 d, *P* = 0.009), and they also had dramatically higher rates of pulmonary infection (50.0% vs 7.1%, *P* = 0.000), multiple organ dysfunction syndrome (MODS) (25.0% vs 5.3%, *P* = 0.041), gastrointestinal complications (25% vs 5.3%, *P* = 0.041) and tracheotomy (33.3% vs 8.0%, *P* = 0.022) after surgery. However, no significant differences were observed in the non-neurological complications, including reoperations for bleeding, heart dysfunction, myocardial infarction, lethal arrhythmia, renal insufficiency, wound infection, or sepsis between the patients in these two groups. The rates of the use of extracorporeal membrane oxygenation (ECMO) were confirmed to be approximately equal between the two groups (Table 3).

**Table 3:** Short term outcomes and hospital costs

Category	PCC (+) *n* = 12	PCC (−) *n* = 113	*P*
Anesthesia recovery period (h)^a^	19.5 ± 33.0	10.2 ± 4.9	0.072
New onset stroke	2(16.7%)	0(0.0%)	/
Syncope	1(8.3%)	0(0.0%)	/
POD	2(16.7%)	0(0.0%)	/
POCD	3(25.0%)	0(0.0%)	/
DEA	2(16.7%)	0(0.0%)	/
Coma	2(16.7%)	0(0.0%)	/
Total of cerebral complications	12(100.0%)	0(0.0%)	/
Paraplegia	1(8.3%)	1(0.9%)	0.183
Reoperation for bleeding	0(0.0%)	2(1.8%)	1.000
Heart dysfunction ^b^	0(0.0%)	7(6.2%)	1.000
Myocardial infarction	0(0.0%)	2(1.8%)	1.000
Lethal arrhythmia	0(0.0%)	1(0.9%)	1.000
Renal insufficiency^c^	1(8.3%)	12(10.6%)	1.000
Pulmonary infection	6(50.0%)	8(7.1%)	0.000
Gastrointestinal complications^d^	3(25.0%)	6(5.3%)	0.041
Wound infection	0(0.0%)	2(1.8%)	1.000
Sepsis	1(8.3%)	5(4.4%)	0.461
ARDS	1(8.3%)	3(2.7%)	0.336
MODS	3(25.0%)	6(5.3%)	0.041
ECMO assistance	0(0.0%)	2(1.8%)	1.000
Thoracic drainage^e^	534.2 ± 435.9	471.9 ± 504.3	0.574
Intubation time(h)	69.3 ± 28.8	33.3 ± 24.2	0.000
Tracheotomy	4(33.3%)	9(8.0%)	0.022
Length of ICU stay(h)	127.3 ± 72.0	63.5 ± 51.3	0.001
Length of hospital stay(d)	32.0 ± 16.7	21.2 ± 13.0	0.009
Mortality in hospital	1(8.3%)	3(2.7%)	0.336
Mortality after discharge	1(8.3%)	0(0.0%)	0.096
Mortality after surgery^f^	2(16.7%)	3(2.7%)	0.072
Hospital costs (RMB)	272,911.0 ± 60,495.8	224,651.5 ± 61,219.9	0.015

Patients from the PCC(+) group were observed to have experienced significantly longer durations of intubation times, ICU stays, and postoperative hospital stays, and they also had dramatically higher rates of pulmonary infection, MODS and tracheotomy after surgery. The postoperative mortalities of the PCC(+) group had a trend of increasing, but there were no significant differences between the patients in these two groups. Patients from the PCC(+) group spent more money compared with the patients in the PCC(−) group

*PCC* Postoperative cerebral complications, *POD* Postoperative delirium, *POCD* Postoperative cognitive dysfunction, *DEA* Delayed emergence from anesthesia, *ARDS* Acute respiratory distress syndrome, *MODS* Multiple organ dysfunction syndrome, *ECMO* Extracorporeal membrane oxygenation

^a^two patients with postoperative comas were not enrolled in the analysis of recovery times; ^b^ severe heart failure reached NYHA grades III-IV; ^c^ required renal replacement therapy; ^d^ included meteorism, nausea, vomiting, abdominal pain, diarrhea, constipation, and gastrointestinal hemorrhage; ^e^ within 48 h after surgery; ^f^ up to the end of the follow-up period

Five cases of death (5/125, 4.0%) were observed in this study, including 4 cases that occurred in the hospital and 1 case that occurred after discharge. Three of these deaths were attributed to multiple organ dysfunction syndrome (MODS) and 2 were attributed to low cardiac output syndrome (LCOS). Fisher’s exact test showed that there was no significant difference in the mortalities of the patients in the two groups, but we observed an ascending trend in mortality after the surgeries in the patients of the PCC (+) group compared with the patients in the PCC (−) group (16.7% vs 2.7%, *P* = 0.072). Wilcoxon rank sum tests also showed that patients in the PCC (+) group spent remarkably more money than the patients in the PCC (−) group (272,911.0 ± 60,495.8 RMB vs 224,651.5 ± 61,219.9 RMB, *P* = 0.015) (Table 3).

### The results of multivariate logistic regression analysis

A total of 6 variables including age, history of the cerebral diseases, Euro SCORE II, duration of CPB, duration of MHCA+SCP and average rcSO_2_ bilateral baseline were analyzed in the stepwise logistic regression model to identify the independent risk factors for PCC. The multivariate logistic regression analysis showed that the duration of MHCA+SCP and the average baseline value of rcSO_2_ from the bilateral brain before anesthesia induction were the independent risk factors for PCC (OR: 9.009, *P* = 0.034 and OR: 0.080, *P* = 0.009) (Table 4).

**Table 4:** Multivariate logistic regression analysis

Risk factors	B	S.E.	Wald	df	Sig.	Exp(B)
Age	0.454	0.652	0.484	1	0.486	1.574
History of cerebral diseases	−7.529	23.720	0.101	1	0.751	0.001
Euro SCORE II	1.376	0.704	3.822	1	0.051	3.958
CPB (min)	0.498	0.518	0.925	1	0.336	1.646
MHCA + SCP (min)	2.198	1.038	4.483	1	0.034	9.009
Avg rcSO_2_ at baseline^a^	−2.527	0.963	6.889	1	0.009	0.080

The duration of MHCA+SCP and the average value of rcSO_2_ at baseline were ultimately identified as significant risk factors (OR: 9.009, *P* = 0.034 and OR: 0.080, *P* = 0.009)

*CPB* Cardiopulmonary bypass, *MHCA* Moderate hypothermic circulatory arrest, *SCP* Selective cerebral perfusion, *rcSO*_*2*_ regional cerebral oxygen saturation

^a^the average baseline value of rcSO_2_ from the bilateral brain before anesthesia induction

### Follow-up

Three patients were lost within the 12-month follow-up period and were excluded from this study. One incidence of death after discharge was observed in the PCC (+) group and that was attributed to recurrence of the aortic dissection.

## Discussion

Although the technology of cerebral protection has been improved in the last few decades, the morbidity of PCC after surgery for AAD occurs at such a high frequency that we cannot ignore the harmful impact of it. In this study, patients with PCC had dramatically higher rates of pulmonary infection and tracheotomy after surgery, and this was relevant with the longer duration of postoperative intubation. Considering the impaired mental state and the poor spontaneous respiration of the patients, the physicians in the ICU prefer the prolonged assistance of mechanical ventilation in these patients after the surgeries for AAD. Therefore, it is almost inevitable that the incidences of postoperative ventilator-associated pneumonia (VAP) and tracheotomy would increase.

A prolonged duration of mechanical ventilation assistance would certainly result in a prolonged ICU and hospital stays. A longer duration of ICU stay and ventilatory support would lead to a higher risk of multiple drug-resistant bacterial infection and multiple organ dysfunction. In addition, it has been reported that the incidence of MODS in patients with severe stroke reached up to 34% [12]. Moreover, more elderly patients were observed in the PCC (+) group, which could be another risk factor for postoperative MODS. Therefore, it made sense that patients with PCC had a high rate of MODS.

A higher incidence of gastrointestinal complications was also observed in the patients in the PCC (+) group after surgery in this study. The probable reasons are: 1. Neuromodulatory dysfunction as a result of cerebral ischemia, which could impair the hypothalamic-pituitary-adrenal axis and the sympathetic nervous system and finally destroy the gastrointestinal barrier function [13–15]; 2. Postoperative therapy of antiplatelet agents, anticoagulants and steroids, which are frequently applied to the patients after surgery for AAD; 3. Pulmonary infection, which was reported to be associated with development of gastrointestinal bleeding after acute stroke [16]; 4. Older age, which is another important risk factor for upper gastrointestinal bleeding according to one report [17]. Furthermore, because of the higher mortality due to pulmonary infection, MODS and GI complications which dramatically prolong the duration of ICU and hospital stay, the patients with PCC logically spent more money in hospital compared with the patients without PCC.

The survival rates of the patients after the surgeries between the two groups had no significant differences at the time of hospitalization and discharge. Nevertheless, we could find an increasing trend in mortality rates of the patients in the PCC (+) group. Therefore, we speculated that PCC plays an important role in the prognosis of patients after surgery for AAD, and this could be attributed to the high rates of postoperative pulmonary infection, gastrointestinal complications and MODS in the patients with PCC. Therefore, it is vital to evaluate the risk factors for PCC in these patients and to provide a scientific basis for an effective preventive strategy.

As we know, the main mechanism of postoperative cerebral dysfunction is an imbalance of oxygen metabolism in the brain which results from microemboli, hypothermia, hypoperfusion, hyperglycemia, reperfusion injury (RI), narcotics and internal environment disturbance. Moreover, a majority (73%) of the patients in China were reported to be affected by a lack of an integral circle of Willis [18], and the intracranial artery atherosclerosis caused by uncontrolled hypertension, hyperglycemia, hypercholesterolemia, and obesity, which frequently occur in elderly patients and patients with a history of cerebral disease, may further impair the insufficient cerebrovascular cross-circulation [19–21]. Therefore, SCP will not meet the oxygen demands of the different regions of the brain.

The analysis in this study confirmed that the age, history of the cerebral disease, duration of CPB, duration of HCA + SCP and rcSO_2_ baseline were the potential risk factors of PCC during the preliminary statistical analysis in this study. Moreover, the multivariate logistic regression analysis revealed that the duration of MHCA+SCP and the average baseline value of rcSO_2_ from the bilateral brain before anesthesia induction were the independent risk factors for PCC. This indicated that anoxia, hypothermia, hypoperfusion and RI could conceivably play significant roles in the occurrence of PCC. Since the branches of the aortic arch are frequently implicated, the low value of rcO_2_ at baseline was considered to be a part of the systematic malperfusion in the patients with AAD. It was reported that a low value of rcO_2_ at baseline is an important predictive factor for the prognosis of patients who undergo cardiovascular surgeries. Although the rcO_2_ at baseline has a weak relationship (OR = 0.080) with PCC in our study, surgeons and anesthesiologists should pay more attention to patients with a low value of rcO_2_ before induction. Currently, it is nearly impossible to doubt that cerebral malperfusion during the circulatory arrest seriously damages the function of the central nervous system (CNS) and the RI following the MHCA will make the damage worse because of the widely disseminated inflammation in the brain. To alleviate ischemic injury, the surgeons always try their best to shorten the duration of circulatory arrest by modifying the surgical procedures and reducing the patient’s core body temperature to an appropriate range. Eight years ago, our department first reported a novel surgical procedure, the triple-branched stented graft implantation, which could be applied to cure AAD with a relatively short duration of systemic circulatory arrest and without a large amount of intraoperative blood loss [22].

However, there are some keypoints which should not be ignored by surgeons: first, the hypothermia itself and CPB methods may depress the regulatory functions of blood flow in the brain and may induce cerebral ischemia during the process of the SCP. A normal level of perfusion pressure or blood flow rate may mask the malperfusion of the CNS [23]; second, in addition to the side effects of the anesthetics, intraoperative stress, and internal environment imbalance, we should also consider the disadvantage of the monitoring of rcSO_2_ in that this monitoring only focuses on the imbalance of oxygen metabolism in the bilateral frontal lobe, and it is not sensitive to the abnormal statuses in the other regions of the brain outside of the frontal lobe. It would be much more appropriate to use a whole-brain oxygen saturation monitoring method or a real-time detection of the bilateral cerebrovascular blood flow by transcranial Doppler sonography (TCD) during the surgery [24]; and Third, cerebral hyperperfusion should be avoided.

Intraoperative monitoring of rcSO_2_ was traditionally considered to be a fairly reliable forecasting and preventive measure of PCC, and in this study, the average value of rcSO_2_ baselines was found to be significantly lower in the patients with PCC compared with the patients in the PCC(−) group. However, the same tendency of rcSO_2_ did not occur during the surgeries in this study. Does that mean that the monitoring of rcSO_2_ during the aortic surgeries is unnecessary? Obviously, the answer is no. In the course of aortic surgeries, we have observed several dramatic drops of rcSO_2_, and an emergency plan was initiated immediately to deal with the anoxia in the brain. Afterwards, the cerebral oxygenation would greatly improve within 5~10 min for approximately 70% patients who underwent aortic surgeries. This revealed that the intraoperative monitoring of rcSO_2_ could provide a much earlier warning indication of cerebral hypoxia comparing with conventional monitoring methods. Therefore, rcSO_2_ is considered to be an essential monitoring means during surgeries for AAD.

This study has several limitations. First, this retrospective case-control and single-center study could not ensure that the patients from the two groups received uniform therapeutic strategies. The selection bias from the subjective judgment of the physicians should not be ignored. Second, due to the relatively small sample size, the absolute quantity of the patients with PCC was relatively small and might reduce the statistical efficiency. Third, we could not exclude the possibility of underestimated mortalities, which were attributed to the relatively short duration of the follow-up period. Therefore, larger sample sizes and more randomized, controlled tests are required for further validation of these procedures.

## Conclusions

PCC has a serious influence on the prognoses of patients who undergo surgical treatment for AAD. The duration of MHCA+SCP and the average baseline value of rcSO_2_ from the bilateral brain before anesthesia induction were the risk factors for PCC. Surgeons and anesthesiologists should pay more attention to the patients with low values of rcO_2_ before induction, and shortening the length of MHCA+SCP may be an effectively prophylactic measure to reduce the morbidity of PCC in the patients with AAD. Larger sample sizes and more randomized, controlled tests are required for further validation of these procedures.

## Data Availability

The datasets generated and/or analysed during the current study are not publicly available due to the confidentiality agreement of our institution but are available from the corresponding author on reasonable request.

## Abbreviations

AAD: Stanford type A aortic dissection
AMI: Acute myocardial infarction
ARDS: Acute respiratory distress syndrome
ASA: American Society of Anesthesiologists
AUC: Area under the curve
BIS: Bispectral index
BMI: Body mass index
CAD: Coronary artery disease
CAM-ICU: Confusion assessment method for the intensive care unit
CNS: Central nervous system
CP: Cryoprecipitation
CPB: Cardiopulmonary bypass
DEA: Delayed emergence from anesthesia
ECMO: Extracorporeal membrane oxygenation
EF: Ejection fraction
FFP: Fresh frozen plasma
GCS: Glasgow Coma Scale
LCOS: Low cardiac output syndrome
MAP: Mean arterial pressure
MHCA: Moderate hypothermic circulatory arrest
MODS: Multiple organ dysfunction syndrome
NIHSS: National Institutes of Health Stroke Scale
NYHA: New York Heart Association
PCC: Postoperative cerebral complications
PEEP: Positive end-expiratory pressure
PetCO_2_: End tidal CO_2_ volumes
PLT: Platelet
POCD: Postoperative cognitive dysfunction
POD: Postoperative delirium
RASS: Richmond Agitation Sedation Scale
RBC: Red blood cell
rcSO_2_: regional cerebral oxygen saturation
RI: Reperfusion injury
SCP: Selective cerebral perfusion
TCD: Transcranial Doppler sonography
TEE: Transesophageal echocardiography
UCG: Ultrasound cardiogram
VAP: Ventilator-associated pneumonia
